# Dissecting Antibodies with Regards to Linear and Conformational Epitopes

**DOI:** 10.1371/journal.pone.0121673

**Published:** 2015-03-27

**Authors:** Björn Forsström, Barbara Bisławska Axnäs, Johan Rockberg, Hanna Danielsson, Anna Bohlin, Mathias Uhlen

**Affiliations:** 1 Science for Life Laboratory, KTH—Royal Institute of Technology, SE-171 21 Stockholm, Sweden; 2 Department of Proteomics, School of Biotechnology, AlbaNova University Center, Royal Institute of Technology (KTH), Stockholm, Sweden; New York State Dept. Health, UNITED STATES

## Abstract

An important issue for the performance and specificity of an antibody is the nature of the binding to its protein target, including if the recognition involves linear or conformational epitopes. Here, we dissect polyclonal sera by creating epitope-specific antibody fractions using a combination of epitope mapping and an affinity capture approach involving both synthesized peptides and recombinant protein fragments. This allowed us to study the relative amounts of antibodies to linear and conformational epitopes in the polyclonal sera as well as the ability of each antibody-fraction to detect its target protein in Western blot assays. The majority of the analyzed polyclonal sera were found to have most of the target-specific antibodies directed towards linear epitopes and these were in many cases giving Western blot bands of correct molecular weight. In contrast, many of the antibodies towards conformational epitopes did not bind their target proteins in the Western blot assays. The results from this work have given us insights regarding the nature of the antibody response generated by immunization with recombinant protein fragments and has demonstrated the advantage of using antibodies recognizing linear epitopes for immunoassay involving wholly or partially denatured protein targets.

## Introduction

Antibodies are invaluable tools for the study of proteins and antibody-based methods are widely used both in research and diagnostics [1]. In addition, antibodies have become one of the largest growing fields in therapeutics with a large number of antibody biopharmaceuticals introduced into the clinic during the last years for treatment of e.g. cancer, autoimmunity and inflammation [2,3]. The number of publicly available antibodies is growing with a fast pace as exemplified by the fact that there are more than a million antibodies available towards human targets as part of various antibody listing sites, such as the Antibodypedia portal [4] www.antibodypedia.com.

A key to understanding the varying performance of antibodies is to determine the binding sites, or epitopes, they recognize. Epitopes are generally divided in two categories, linear epitopes where a stretch of continuous amino acids are sufficient for binding and conformational epitopes where key amino acid residues are brought together by protein folding [5]. Conformational epitopes might be preferred for applications involving protein targets in their native state, such as therapeutic applications or flow cytometry. On the other hand, linear epitopes might be preferred for applications in which the protein target is wholly or partially denatured during the sample preparation prior to the immuno assay, such as in Western blot (WB), immunohistochemistry (IHC) or immunofluorescence-based confocal microscopy.

The choice of immunogen used for immunization can greatly influence the performance of the resulting antibodies. Immunogens vary from short peptides of only 10–20 amino acids coupled to carrier proteins [7,8], longer protein fragments [9] and up to using the entire full-length proteins [10]. Using full-length proteins for generation of antibodies has the drawback of possibly creating antibodies that are cross-reactive to other proteins sharing sequence similarities with the protein target. Using short peptides or protein fragments can be a way to circumvent this problem, since they can be chosen to cover a unique part of the amino acids sequence. However, anti-peptide antibodies often lack the ability to bind the native proteins, due to the unstructured nature of the peptide [11]. An attractive alternative is therefore to generate antibodies towards protein fragments covering 50–150 amino acids of a unique sequence region of the target protein as compared to other proteins from this species [9]. This strategy of using recombinant protein fragments, referred to as Protein Epitope Signature Tags (PrESTs), as immunogens have been used to generate more than 16,000 validated polyclonal antibodies towards human protein targets within the framework of the Human Protein Atlas (www.proteinatlas.org) project [12,13]. The aim of this study was to characterize polyclonal sera from immunizations with the above-mentioned recombinant protein fragments to determine if this strategy evokes an antibody response targeting mainly linear or conformational epitopes.

Here, we describe how epitope-specific fractionation of polyclonal sera based on epitope mapping and affinity capture on chromatography columns can be used to determine the ratio of antibodies targeting linear and conformational epitopes. This approach also allowed us to investigate the performance of antibody fractions targeting linear and conformational epitopes in the most frequently used immunoassay in life science, namely Western blot.

## Methods

### Antigen production and immunization

The software PRESTIGE [14] was used to design protein fragments, 95–149 amino acids long, with low sequence similarity to other proteins. Gene fragments were amplified from a pool of human RNA and cloned into an *Escherichia coli* vector. The antigens were expressed as fusions of a His_6_-ABP tag and the protein fragments, purified on IMAC columns, validated and used for immunization of New Zeeland White rabbits as described elsewhere [15].

### Epitope mapping

N-terminally biotin-tagged overlapping synthetic 15-mer peptides with five amino acids lateral shift (PEPscreen, Sigma–Aldrich, St Louis, MO) covering the antigen sequences were dissolved in 80% DMSO to a concentration of 10 mg/ml. An aliquot of 2 μL was diluted in 198 μL PBS-B (1×PBS, 1% BSA, pH 7.4) and the rest was stored at -80°C until further use. Color-coded microsphere (MagPlex Microspheres, Luminex-Corp., Austin, TX) previously coated with Neutravidin (Pierce, Rockford, IL), according to the manufacturers instructions, were distributed in a half-area 96-well plate (approx. 19000 per bead-ID) and diluted peptides were added to a final concentration of 50 μg/mL in 100 μL PBS-B. The plate was sealed and incubated for 1 hour on a plate shaker in the dark and then washed three times with 100 μL PBS-T (1×PBS, 0.1% Tween20, pH 7.4) using a magnetic microplate washer (BioTek, Winooski, VT) before re-suspending the beads in 50 μL PBS-T. Re-suspended beads with peptides corresponding to the same antigens were pooled to create the different bead arrays and they were stored in storage buffer (BRE, Blocking Reagent for ELISA, Roche supplemented with 0.1% Pro-clean 400). Polyclonal antibodies and epitope specific antibody fractions were diluted in PBS-B to 1 μg/mL or 0.1 μg/mL and 100 μL were incubated with bead mix corresponding to approximately 1000 beads per bead-ID in a half-area 96-well plate (Greinier, Kremsmünster, Austria) for 1 hour on a plate shaker in the dark. After primary incubation the plate was washed three times with PBS-T before 100 μL secondary antibody (R-phycoerythrin-labeled anti-rabbit IgG antibody (Jackson ImmunoResearch Laboratories, USA)) diluted to 1ng/mL in PBS-B was added to all wells. The plate was incubated for 1 hour on a plate shaker in the dark and then washed three times with PBS-T before the beads were suspended in 50 μL PBS-T and analyzed using a Luminex FLEXMAP 3D (Luminex-Corp., Austin, TX). The median fluorescence intensities of each bead-ID were used to determine the binding to the linear epitopes present on the overlapping peptides.

### Affinity fractionation

**Fig 1:**
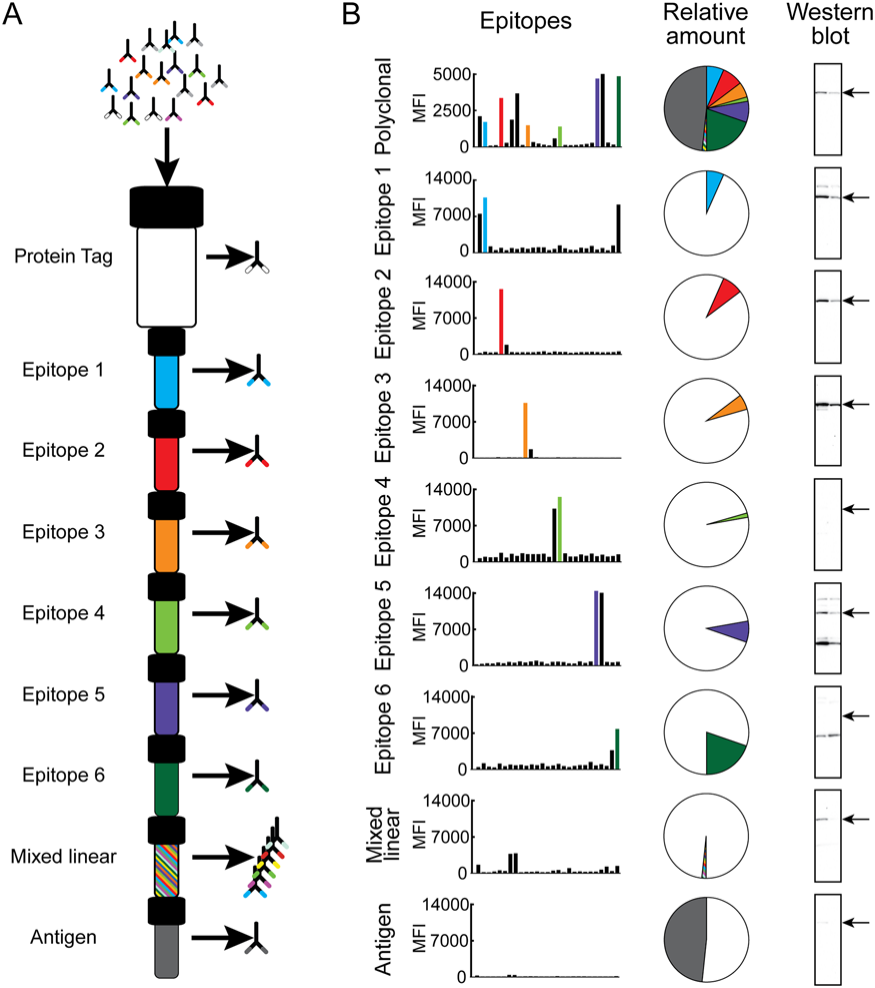
Fractionation of polyclonal antibodies. (A) Illustration depicting the scheme for decomposition of polyclonal antibodies into fractions targeting conformational and linear epitopes. First columns containing the antigen’s protein tag, peptides corresponding to previously mapped linear epitopes, a mix of overlapping peptides covering the antigen sequence and the antigen used for immunization are serially connected. Polyclonal serum is run through the columns where anti-tag antibodies are depleted by the first column, the peptide columns capture antibodies targeting the different linear epitopes and the antigen column binds the remaining antibodies that are targeting conformational epitopes. The columns are then separated from each other and the different antibody fractions are eluted in parallel. (B) An example of results from validation of a polyclonal antibody and antibody fractions towards the target protein tryptophanyl-tRNA synthetase. The left column shows epitope mapping confirming the peptide specificity of the different fractions. The middle column shows the relative antibody amount in each fraction. The right column shows the ability of each antibody fraction to detect a band of expected molecular weight, indicated by an arrow, in Western blots assays with RT-4 and U-251 MG lysates.

Six hundred nanomoles of the biotinylated peptides corresponding to the major epitopes of the polyclonal sera was applied to 1 mL HiTrap Streptavidin HP columns (GE Healthcare Bio-Sciences AB, Uppsala, Sweden) for binding. For each target a mixed peptide column was also created by pooling all peptides corresponding to the antigen before they were applied to the column. Approximately, 8–12 mL of sera was purified on a ÄKTAxpress (GE Healthcare) liquid chromatography system on respective columns in a serial manner as indicated by Fig 1A. After sample loading, the columns were washed and eluted in parallel to obtain separate monospecific antibody fractions and a fraction from the antigen column. To verify the monospecificity, the eluted fractions were epitope mapped as described above. The antibody concentration in each eluted fraction was determined by spectrophotometric measurement at 280 nm.

### Western blot

The antibody fractions were analyzed on Western blot membranes prepared from 15 μg of total protein lysates from one or more of the three cell lines (A431, RT-4 and U-251 MG), two tissues (liver and tonsil), plasma and HEK293 cells over-expressing the target protein CRABP2 (LY419677, Origene Technologies). Molecular weight marker PageRuler (Pierce, Rockford, IL) and lysates were loaded on precast 10–20% Criterion SDS-PAGE gradient gels (Bio-Rad Laboratories, Hercules, CA) and separated according size under reducing conditions. The proteins were then transferred to PVDF membranes (Bio-Rad Laboratories, Hercules, CA) using Criterion Gel Blotting Sandwiches (Bio-Rad Laboratories, Hercules, CA) according to the manufacturer’s instructions and the membranes were blocked (5% dry milk, 0.5% Tween-20, 1 x TBS; 0.1M Tris-HCl, 0.5M NaCl) for 1 h at RT. The polyclonal antibodies were diluted 1/250 in blocking buffer and the epitope specific polyclonal antibodies were normalized to match the antibody concentration of their respective polyclonals. After 1h incubation the membranes were washed 4 x 5 minutes in TBS-T (1xTBS, 0.05% Tween20). Secondary HRP-conjugated swine anti-rabbit antibody (DakoCytomation, Glostrup, Denmark) was diluted 1/3000 in blocking buffer and the membranes were incubated 45 min. The membranes were again washed 4 x 5 min in TBS-T before addition of HRP-substrate (SuperSignalVR West Dura Extended Duration Substrate, Pierce) and chemiluminescence detection was carried out using a ChemiDoc CCD-camera system (Bio-Rad Laboratories).

### Ethics statement

This study was carried out in strict accordance with applicable rules, regulations and professional standards relating to the use of animals for scientific purposes advised by the Swedish Board of Agriculture. In this study, immunizations of New Zealand White rabbits were performed according to a protocol approved by the Umeå ethical board of animal studies of the Court of Appeal for Upper Norrland (Swedish permit no A80-05 for AgriSera AB, Vännäs, Sweden). All terminal bleedings by cardiac puncture were performed under Hypnorm anesthesia, and all efforts were made to minimize suffering. Tissue and liver samples for Western blot assays were obtained from a biobank of fresh frozen tissue samples. Tissue samples in the biobank were collected, after informed consent, from patients who had undergone surgery for diagnostic or therapeutic reasons. The local ethics committee at Uppsala University Hospital provided an advisory statement that there are no ethical objections to utilizing material from the biobank for antibody validation, provided that the material is anonymized.

## Results

### The principle of the study

The aim of this study was to determine the proportions of antibodies towards linear and conformational epitopes elicited by immunizations with recombinant protein fragments. This was accomplished by using an approach described by Hjelm et al. [16] for the purification of epitope-specific antibodies as illustrated in Fig 1A. First linear epitopes of antiserum, obtained from immunization of rabbits with recombinant protein fragments of approximately 100 amino acid residues, were identified using overlapping 15-mer peptides coupled to color-coded microspheres in a suspension bead array [17]. Identified epitope peptides were coupled to liquid chromatography columns and serially connected to columns containing the antigen, protein tag and a mix of all 15-mer peptides from the epitope mapping. Polyclonal serum was then flowed through the serially connected columns, first capturing antibodies targeting the protein tag, then the antibodies targeting linear epitope peptides and last antibodies targeting conformational epitopes were captured by the antigen column. All columns were then separated and the individual antibody fractions were eluted in parallel.

### Dissecting antibodies towards human protein WARS

Fig 1B concludes the validation of the polyclonal sera against tryptophanyl-tRNA synthetase (WARS) and its resulting fractions after affinity purification against linear and conformational epitopes. Six peptides (marked in colors) were chosen from the mapping of the polyclonal serum and used as ligands to generate six epitope specific fractions. The fractions were mapped using the same peptide set as for the polyclonal serum to determine the specificity towards a single epitope for each fraction (“Epitopes”). The results confirm that each epitope fraction detects its respective epitope peptide and that the antigen fraction shows no binding to any linear epitopes. The antibody amount in each fraction was determined using spectrophotometric absorbance measurement at 280 nm and the relative amounts of the affinity-purified fractions are shown in the pie charts. The largest individual fraction of antibodies in the polyclonal serum was in this case directed towards conformational epitopes, while the most dominant linear epitope was situated C-terminally on the antigen sequence (epitope 6). The antibody fractions were validated in Western blot analysis of RT-4 and U-251 MG lysates and were defined as positive in Western blot if they showed staining of a band of correct size (53 or 48 kDa), and negative if showing no binding or off-target binding to bands of other sizes. The results show that the polyclonal pool of antibodies recognizes a band of the correct size and this is also observed for epitope-specific antibodies towards epitopes 1, 2, 3 and 5. In contrast, no reactivity towards protein of correct size is observed for antibody fractions towards epitope 4 and 6. Off-target binding is observed in fractions against epitope 1, 5 and 6 and only a faint band of correct size is detected for the antibodies towards the conformational epitopes (antigen). The results demonstrate a large variation in immunological reactivity of the epitope-specific antibodies with only some recognizing the target protein in Western blots, while the other either show no binding to the target and/or off-target binding. Note that the positive epitope-specific antibodies with no or little off-target binding (epitope 1,2 and 3) represents less than 25% of the antibodies in the original polyclonal antisera and thus more than 75% of the antibodies are shown to be negative in the Western blot assay, although all antibodies have been purified by specific capture to the protein fragment antigen. Despite this, the original polyclonal mixture with only 25% of the antibodies binding a band of correct molecular weight yields excellent Western blot results with little off-target binding.

### Antigens and their resulting epitope properties

**Fig 2:**
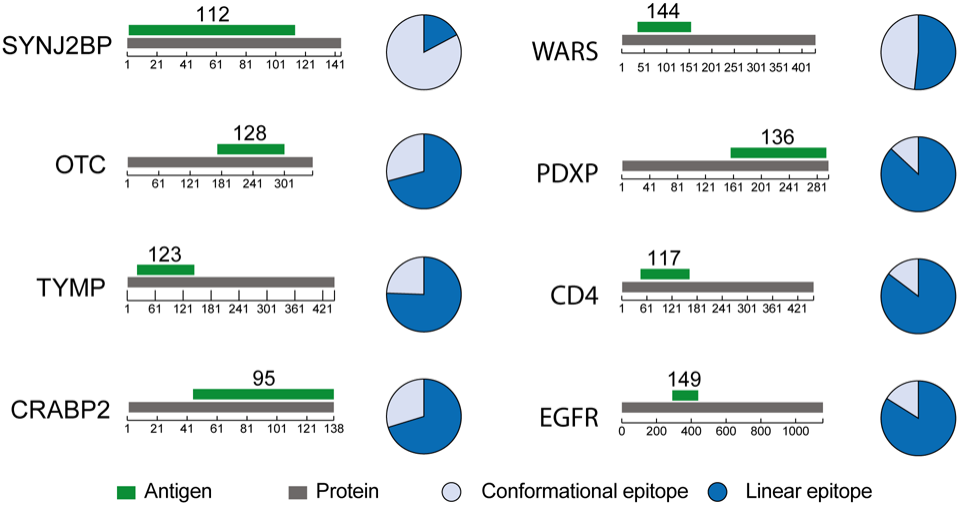
Proportion of antibodies towards linear and conformational epitopes in eight polyclonal sera. Each protein target is represented in grey with the amino acid length indicated under the bar. Antigens used for immunization are shown above in green with the amino acid lengths shown above. Pie charts to the right of each protein show the relative amount of purified antibodies toward conformational (light blue) and linear (dark blue) epitopes.

We decided to investigate eight polyclonal antibodies generated towards human recombinant protein fragment (PrESTs) with the lengths of the eight antigens varying between 95–149 amino acids. The sizes (number of amino acids) and locations sizes for the eight antigens on their respective full-length protein sequence are shown in Fig 2 together with pie charts showing the fractions antibodies targeting linear and conformational epitopes in the polyclonal sera. For six of the protein targets (OTC, TYMP, CRABP2, PDXP, CD4 and EGFR), a large majority (70–90%) of the antibodies recognize linear epitopes, while for one of the targets (SYNJ2BP) the majority (80%) of the antibodies are shown to bind conformational epitopes. For WARS, already described above, approximately 50% of the antibodies are linear and conformational, respectively. The results suggest that most of the epitopes generated in this manner yield antibodies recognizing linear epitopes.

### Western blot performance of the epitope-specific antibodies

**Fig 3:**
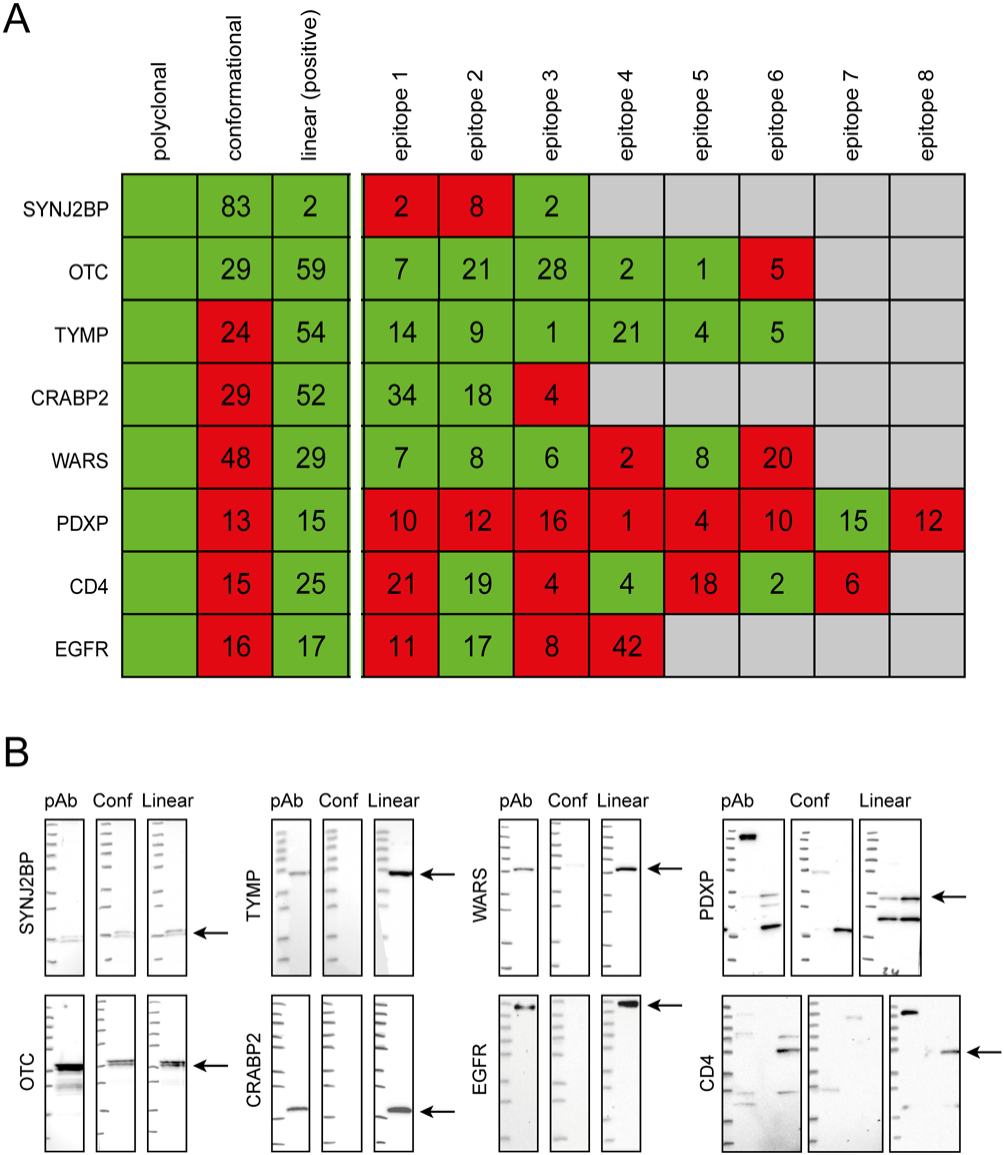
Western blot performance of antibody fractions. (A) The ability of the different antibody fractions to detect a protein of the expected molecular weight in Western blot is shown in green (positive) or red (negative). The number indicates the relative amount in percent of each antibody fraction and a total of antibody fractions towards linear epitopes that are able to bind the target protein in Western blot. (B) Western blots analysis of eight different protein targets. Detection of protein targets using polyclonal antibody, antibody fraction towards conformational epitopes and one representative antibody fraction towards a linear epitope. Expected molecular weights are indicated by arrows on the right of each set of Western blots. Lysate panels for the target proteins: SYNJ2BP (Marker, RT-4), OTC (Marker, liver), TYMP (Marker, liver), CRABP2 (Marker, LY419677), WARS (Marker, RT-4, U-251 MG), PDXP (Marker, RT-4, liver), CD4 (Marker, U251-MG, plasma, tonsil), EGFR (Marker, A431).

The relative amounts, epitope specificity and Western blot performance of the epitope-specific antibodies were further investigated using spectrophotometric absorbance measurement, suspension bead array assay with overlapping peptides as described above and Western blot assays, respectively. The results are summarized in Fig 3 showing the fraction (%) of antibodies towards linear and conformational and the antibodies towards linear epitopes further stratified towards all the epitopes ranging from three linear epitopes (SYNJBP and CRABP2) to eight epitopes (PDXP). Each fraction was analyzed in Western blot assays as shown in Fig 3B, and the results are summarized in Fig 3A as positive in Western blot (green) defined as staining a band of correct size and negative (red) if no staining or off-target binding. The Western blots (Fig 3B) show examples of the polyclonal antibody, conformational antibody fraction and one linear antibody fraction towards each target protein. The results demonstrate that for all eight target proteins at least one fraction towards linear epitopes recognizes the protein in Western blot, although in the case of SYNJ2BP this fraction corresponds to only a few percent of the total amount. The antibodies toward conformational epitopes were not only generally less abundant, but also showed limited ability to bind the target protein in Western blot, where only two out of the eight fractions showed a band of correct molecular weight.

### The structure of the epitopes

**Fig 4:**
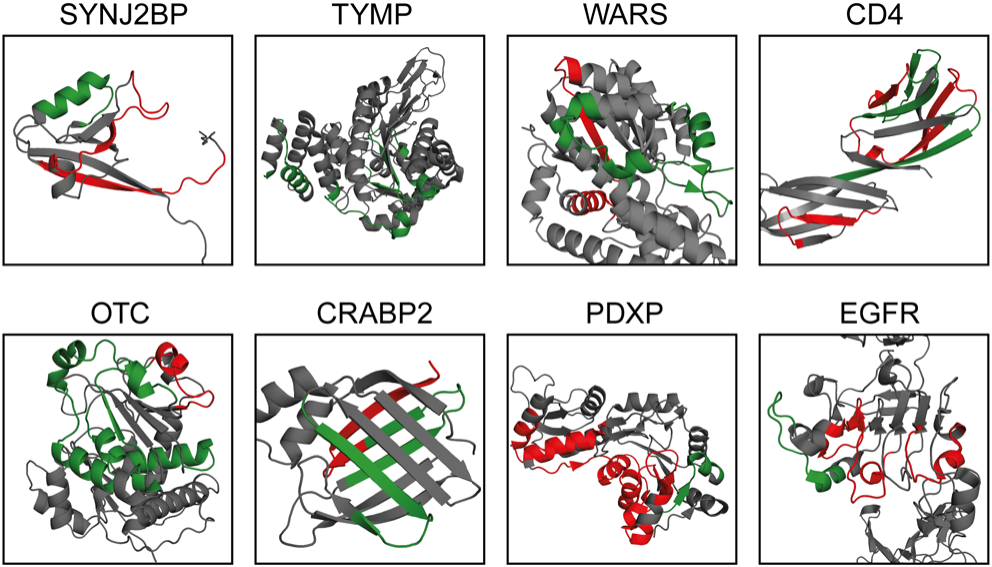
Structure analysis of epitopes. 3D structures of protein targets with highlighted linear epitopes in green where the corresponding antibody fraction showed bands of correct molecular weight in Western blot analysis and in red where the antibody fraction did not bind the target protein. SYNJ2BP (2eno.pdb), TYMP (2jof.pdb), WARS (1r6t.pdb), CD4 (1wio.pdb), OTC (1oth.pdb), CRABP2 (2g7b.pdb), PDXP (2cft.pdb) and EGFR (3njp.pdb).

The three-dimensional structures of the eight protein targets investigated in this study had previously been determined and were available in the protein data bank (pdb.org), which made it possible to show the structural nature of each of the epitopes (Fig 4). Since only some of the epitope-specific antibodies were shown to bind the target protein in the Western blot assay, each epitope sequence is color coded green (positive in the Western blot assay) or red (negative). For SYNJ2BP, the Western blot positive epitope is located on an α -helix, while the negative epitopes are parts of a β-strand and a loop-structure. For TYMP and WARS, almost all the epitopes are positive in Western blot and they are all parts of α-helices, while a loop structure in WARS is negative. For CD4, consisting entirely of β-sheets, all epitopes are part of β-strands and some are bind the target protein in Western blot, which is also the case for CRABP2. For OTC, the majority of the epitopes are part of α-helices and they are all Western blot positive. For PDXP, all epitopes are in α-helices, but all except one of these are negative. As for PDXP, only one of the linear epitopes for EGFR is positive and the epitope spans a loop and a part of an α-helix. Interestingly, although immunizations were performed using protein fragments and not full-length proteins, the majority of the linear epitopes positive in Western blot are localized on the surface of the proteins.

## Discussion

In this study, we have dissected the nature of the polyclonal antibody responses generated by immunizations with recombinant protein fragments. For all eight protein targets investigated here, at least three distinct linear epitopes were obtained, which supports recent result from epitope mapping of more than 900 polyclonal antibodies using *in situ* synthesized high-density peptide arrays, showing that these antibodies recognize three linear epitopes on average [18]. In all cases, a fraction of the antibodies also recognized conformational epitopes, here defined as antibodies recognizing the antigen used for immunization, but not binding to any of the overlapping 15-mer peptides covering the antigen sequence. However, for a majority of the analyzed antibodies, the fraction of the antibodies recognizing conformational epitopes was less than 25% suggesting that the immunization scheme used here preferentially generate antibodies towards linear epitopes.

For all eight targets at least one linear epitope was able to detect a band of expected molecular weight in the Western blot assay, while only two of the fractions towards conformational epitopes showed correct binding. In the case of WARS, the anti-conformational epitope fraction contained almost half of the total amount of antibodies, but only a faint signal was detected in Western blot suggesting that most of these conformational epitopes were disrupted during the assay. The Western blot assays demonstrated that for four of the protein targets, at least half of the antibodies generated in the immunization were present in fractions that recognized the target protein in a Western blot, while for the other four, only 15–30% of the antibodies were able to detect a band of the expected molecular weight.

The analysis of the three-dimensional structure of the epitopes identified in this study showed that the linear epitopes can be found in any secondary structure, but that there seems to be a slight preference towards α-helices. An interesting observation is that most of the linear epitopes are present on the surface of the native structure and the question remains if this might be due to a partial folding of the protein fragment during the immunization to make the surface epitopes accessible for the immune system or if the presence of surface epitopes merely reflects the higher antigenicity of amino acids displayed on the surface of the target protein.

Taken together, the results suggest a clear advantage of using antibodies recognizing linear epitopes for assays in which the protein target is either fully or partially denatured. The results also show that most of the antibodies generated by immunization using the protocol described here indeed yield antibodies targeting linear epitopes, although antibodies towards conformational epitopes are also present in the serum. These results support the use of protein fragments as immunogens for immunization to generate polyclonal antibodies, at least as long as the generated antibodies are intended for immunoassays involving partially denatured proteins. It is important to point out that the antibodies recognizing linear epitopes will bind to a specific conformation of the linear epitope [6] and thus it still recognizes a conformational-specific epitope in the case of native proteins. More work is needed to identify the linear and conformational epitope pattern using full-length native proteins instead of protein fragments as immunogens and to what extent the protein fragment strategy is also advantageous for the generation of monoclonal antibodies to yield reagents that are able to detect the target protein across a broad panel of applications. The strategy outlined here with epitope mapping, affinity purification using synthetic peptides and subsequent binding assays is a powerful tool for dissecting the structure and function of the binding of an antibody towards its target protein and the results have given us new insights regarding the nature of the antibody response upon immunizations with protein fragments.
